# The History of Domestication and Selection of Lucerne: A New Perspective From the Genetic Diversity for Seed Germination in Response to Temperature and Scarification

**DOI:** 10.3389/fpls.2020.578121

**Published:** 2021-01-21

**Authors:** Wagdi Ghaleb, Lina Qadir Ahmed, Abraham J. Escobar-Gutiérrez, Bernadette Julier

**Affiliations:** INRAE, URP3F, Lusignan, France

**Keywords:** alfalfa, breeding, genetic variation, *Medicago sativa*, seed dormancy, adaptation

## Abstract

Lucerne (*Medicago sativa*), a major perennial pasture legume, belongs to a species complex that includes several subspecies with wild and cultivated populations. Stand establishment may be compromised by poor germination. Seed scarification, deterioration and temperature have an impact on germination. The objective of this study was to analyse the genetic diversity of lucerne germination in response to three factors: (1) temperature, with seven constant temperatures ranging from 5 to 40°C, was tested on 38 accessions, (2) seed scarification was tested on the same accessions at 5 and 22°C, (3) seed deterioration was tested on two accessions and two seed lots at the seven temperatures. The germination dynamics of seed lots over time was modelled and three parameters were analysed: germinability (germination capacity), maximum germination rate (maximum% of seeds germinating per time unit), and lag time before the first seed germinates. Seed scarification enhanced germinability at both temperatures and its effect was much higher on *falcata* and wild *sativa* accessions. Incomplete loss of the hardseededness trait during domestication and selection is hypothesised, indicating that the introduction of wild material in breeding programmes should be followed by the selection for germinability without scarification. Seed lots with altered germinability had low germination at extreme temperatures, both cold and hot, suggesting that mild temperatures are required to promote germination of damaged seed lots. A large genetic diversity was revealed for germination (both capacity and rate) in response to temperature. All accessions had an optimal germination at 15 or 22°C and a poor germination at 40°C. The *sativa* varieties and landraces had a high germination from 5 to 34°C while the germination of *falcata* and the wild *sativa* accessions were weakened at 5 or 34°C, respectively. These differences are interpreted in terms of adaptation to the climate of their geographical origin regions in order to escape frost or heat/drought risks. These new findings give insights on adaptation and domestication of lucerne in its wide geographic area. They suggest further improvement of germination is needed, especially when introducing wild material in breeding pools to remove scarification requirements and to limit differences in response to temperature.

## Introduction

Lucerne (*Medicago sativa* L.) is a perennial forage legume that holds a major place in cultivated grasslands worldwide. It is used for ruminant feeding, given its high yield and nutritional properties. The numerous ecosystem services it provides (Julier et al., 2017) are important in the present phase of agroecological transition (Lamichhane et al., 2018), and mainly result from its ability to fix atmospheric nitrogen. *M. sativa*, which was first cultivated in its centre of origin (Near-East to Middle-East) 9,000 years ago, is the oldest cultivated forage crop and one of the oldest crops in the world. It spread several times to North Africa and Europe through invaders (Medes, Romans and Moors) and was introduced to Asia (India and China) more than 2,000 years ago (Basigalup et al., 2014). From the 15th Century, lucerne became a popular crop in France and other European countries (De Serres, 1600) from where it accompanied explorers and migrants to America from the 16th Century onwards and to austral countries by the 19th Century (Prosperi et al., 2014). Lucerne is now grown in temperate regions worldwide and has recently became a cornerstone crop in organic agriculture. Its direct annual world value is expected to grow significant over the coming years.

*Medicago sativa* is a complex of several diploid or autotetraploid perennial and allogamous subspecies (ssp), wild or cultivated, that freely intercross at the same ploidy level (Small and Jomphe, 1989). Ssp *sativa* with coiled, non-spiny pods, and purple or purple yellow flowers, includes the vigorous cultivated autotetraploid varieties, some wild populations in Spain but also the diploid wild form *coerulea*. The diploid ssp *glomerata* exhibits coiled pods and yellow flowers. Ssp *falcata* with yellow flowers and slicked-shaped pods, originates from Northern Eurasia and is cold tolerant. The populations that result from hybridisation between ssp *sativa* or *coerulea* and ssp *falcata* are sometimes described as ssp x*varia* (Small and Brookes, 1990). As shown by their variegated flower colour, most European varieties exhibit introgression from ssp *falcata* that confers cold tolerance and has enabled the selection of highly productive varieties adapted to continental or subarctic climates.

As many other annual and perennial legumes, in particular of the *Papilionoideae* subfamily (Baskin and Baskin, 2014; Acosta et al., 2020), it has been well documented that physical dormancy due to hardseededness is present in lucerne to various degrees (Acharya et al., 1999). Hardseededness is characterised by a water-impermeable seed coat that ensures a maintenance of the population in the soil and prevents its germination up to several years (Baskin and Baskin, 2004; Finch-Savage and Leubner-Metzger, 2006). Despite the uncontested ecological advantages associated with the different types of seed dormancy (Baskin and Baskin, 2014), it hampers crop establishment due to low percentage of germinating seeds and slow rates of seedling emergence (Acosta et al., 2020). This type of physical dormancy is broken by different treatments to the seeds, including scarification, which makes imbibition possible by overcoming coat’s impermeability to water (Baskin and Baskin, 2014). Even in cultivated varieties, the presence of hard seeds is common in commercial lots, although regulations and the market impose a minimum percentage of such seeds.

Seed deterioration, often generated by ageing, induces a reduction of germination capacity, or germinability, (Mcdonald, 1999). Evaluation of lot germinability along seed ageing is performed by the seed industry (Corbineau, 2012) and the institutions in charge of *ex situ* seed conservation (Genetic Resources Centres; Fu et al., 2015). Seed deterioration is regulated by seed storage conditions (temperature, humidity and oxygen partial pressure) but also by endogenous factors (Zhou et al., 2020). Little information is available on the impact of temperature on germination of seed lots whose germination capacity is deteriorated.

Temperature is the most important environmental factor regulating both timing and rates of seed germination. The role of temperature in seed germination is related to enzyme activity and membrane permeability, which impact respiratory metabolism, and thus the rate of germination (Bewley and Black, 1994). Plant species have different critical temperature ranges and basic thermal requirement to complete specific phenophases or the whole life cycle (Luo, 2011). Seed germination in response to temperature can be summarised by the cardinal temperatures (minimum, optimum and maximum temperatures; Bewley and Black, 1994; Shafii and Price, 2001; Durr et al., 2015). The response of different species to temperature during germination has been a topical issue for a long time (Bewley and Black, 1994; Baskin and Baskin, 2014; Durr et al., 2015; Tribouillois et al., 2016). Major differences among species depend on the climatic conditions where the species grow or originate. Cultivated species germinate faster and under a wider range of temperatures than wild species, likely as a result of human selection (Durr et al., 2015). Information on genetic variation for germination capacity is useful in breeding programmes especially if wild accessions are used to adapt varieties to new climatic conditions or practices related to ecosystem services, or for the preservation of genetic diversity in genetic resources centres. Studies at the intra-specific level are not frequent in pasture species (Moot et al., 2000; Mcgraw et al., 2003; Monks et al., 2009; Ahmed, 2015; Ahmed et al., 2019). In the model legume species *Medicago truncatula*, a significant diversity for germination was evidenced among accessions (Brunel et al., 2009) and within a mapping population (Dias et al., 2011). Knowledge of physical dormancy, seed deterioration and genetic diversity in response to temperature, is of paramount importance in the management of lucerne breeding programmes.

In this paper, the objective was to demonstrate a genetic diversity of *Medicago sativa* for seed germination in response to constant temperatures (from 5 to 40°C). We performed experiments in controlled conditions with thirty-eight accessions of various geographical origins, from the subspecies *sativa, falcata* or *glomerata*, wild or cultivated. The effects of scarification and deterioration level of seed lots on germination at different temperatures were also tested. All the results were analysed and interpreted in terms of domestication, selection and breeding.

## Materials and Methods

### Plant Material

In this study, seeds of thirty-eight accessions of wild populations, landraces and varieties of lucerne were evaluated as follows: 16 *sativa* varieties, 11 *sativa* landraces, four *sativa* wild accessions, one *falcata* variety, five *falcata* wild populations and one *glomerata* wild population (Table 1). In addition to the spp. membership, the status (wild, landrace or variety) that traces the breeding effort possibly applied to germination traits was used to define five groups of accessions: ‘*falcata* + *glomerata* wild’, ‘*sativa* wild’, ‘*falcata* variety’, ‘*sativa* landraces’ and ‘*sativa* varieties’. Seeds were obtained from INRAE Genetic Resources Centre in Lusignan, France, or from breeders. They were conserved at 5°C and 30% relative humidity until they were used. For two landraces, Flamande from France and Gabès from Tunisia, deteriorated seed lots, characterised by poor germinability, were also included. Only two wild *falcata*, the *falcata* variety and the wild *glomerata* were diploid accessions and all the others were tetraploid accessions, so the effect of ploidy was not studied here.

**TABLE 1:** Description of the 38 accessions of *Medicago sativa*.

						**Geographic coordinates**		
**Code**	**Subspecies**	**Type**	**Ploidy**	**Name**	**Country**	**Longitude**	**Latitude**	**Autumn dormancy**
35	*falcata*	Wild	2x	Romanica	Russia	55.74	37.61	
36	*falcata*	Wild	2x	Quasifalcata	Russia	55.74	37.61	
38	*falcata*	Wild	4x	Krasnokutskaya	Russia	50.91	47.05	
39	*falcata*	Wild	4x	Maron	France	48.63	6.04	
40	*falcata*	Wild	4x	Malzeville	France	48.70	6.18	
37	*glomerata*	Wild	2x	Glomerata	France	43.60	5.75	
20	*sativa*	Wild	4x	Monte Oscuro	Spain	41.72	–0.47	
21	*sativa*	Wild	4x	Villanueva de Jara	Spain	39.44	–1.95	
22	*sativa*	Wild	4x	Villamajor	Spain	41.68	–0.77	
23	*sativa*	Wild	4x	Pancrudo	Spain	40.76	–1.03	
34	*falcata*	Variety	2x	Anik	Canada			1.0
05	*sativa*	Landrace	4x	Flamande*	France	48.85	2.35	4.0
08	*sativa*	Landrace	4x	Poitou	France	46.58	0.34	4.0
09	*sativa*	Landrace	4x	Provence	France	43.53	5.44	6.0
24	*sativa*	Landrace	4x	Gabès*	Tunisia	33.90	10.1	9.0
25	*sativa*	Landrace	4x	Cremonese	Italy	45.13	10.02	6.0
26	*sativa*	Landrace	4x	Crau	France	43.55	4.85	
27	*sativa*	Landrace	4x	Demnate3	Morocco	31.73	–7.00	9.0
28	*sativa*	Landrace	4x	Dra15	Morocco	30.33	–5.83	9.0
29	*sativa*	Landrace	4x	Atlas	Morocco	31.11	–7.87	9.0
30	*sativa*	Landrace	4x	Ziz10	Morocco	31.93	–4.42	9.0
31	*sativa*	Landrace	4x	Baghdadi	Iran	32.13	48.18	9.0
01	*sativa*	Variety	4x	Banat VS	Serbia			5.0
02	*sativa*	Variety	4x	SW Nexus	Sweden			4.0
03	*sativa*	Variety	4x	Luzelle	France			3.0
04	*sativa*	Variety	4x	Holyna	Czech Republic			3.7
06	*sativa*	Variety	4x	Lukal	France			4.0
07	*sativa*	Variety	4x	Ludelis	France			4.0
10	*sativa*	Variety	4x	Barmed	France			7.0
11	*sativa*	Variety	4x	Harpe	France			4.0
12	*sativa*	Variety	4x	Orca	France			4.5
13	*sativa*	Variety	4x	Radius	Poland			
14	*sativa*	Variety	4x	FG-CO416C4164	United States			4.0
15	*sativa*	Variety	4x	Alforex6	United States			4.0
16	*sativa*	Variety	4x	Gongnong1	China			
17	*sativa*	Variety	4x	Magna790	United States/ARG			7.0
18	*sativa*	Variety	4x	Bauding	China			
19	*sativa*	Variety	4x	Picena GR	Italy			7.4

The subspecies, population type (variety, landrace or wild population), name of accession and country of origin are given. For the wild populations, the geographic coordinates of the collection site are indicated. Autumn dormancy is given when known. * Two seed lots of these accessions were tested.

The material was used to test three aspects of seed germination. (1) The effect of scarification, in interaction with genetic diversity, was tested on the whole set of 38 accessions at two temperatures (5 and 22°C). Scarification consisted in scrubbing the seeds between two sheets of sandpaper (grade 150) for 30 s. (2) The effect of the deterioration level of seed lots on germination response to seven constant temperatures (5, 10, 15, 22, 28, 34 or 40°C) was analysed on two accessions by comparing, within each accession, seed lots with high or low germinability. (3) The genetic diversity for germination in response to these seven constant temperatures was evaluated on scarified seeds of the 38 accessions.

### Germination Tests

For each accession and treatment, four repetitions of 100 seeds were prepared. For temperatures between 5 and 34°C, the 100 seeds of each repetition were put in 90 mm diameter Petri dishes containing two sheets of autoclave-sterilised Whatman paper (ref. 3645 Whatman, France) humidified with 5 ml of deionised and autoclave-sterilised water. For the experiment at 40°C, which was conducted in a growth chamber with 80% relative humidity, seeds were placed on two sheets of Whatman papers in transparent crystal polystyrene boxes (180 mm × 120 mm × 50 mm; GEVES trademark, Loire Plastic, France; Brunel et al., 2009) with 15 ml of sterilised water.

For each temperature, the design was a random complete block design (RCBD) with four blocks. The dishes of each block were placed in a vented plastic box. The experiment was conducted in walk-in growth chambers at constant temperature in the dark and with vapour pressure deficit <1 kPa. Temperature of the chambers were checked by six thermocouples placed at different positions within the useful volume, logged every 20 s. Temperature and relative humidity in the chambers were recorded every minute during the germination experiment.

Germination counting was carried out at variable time intervals that depended on temperature treatments: once a day at 5 and 40°C and twice a day for temperatures from 10 to 34°C. Seeds were considered as germinated when the radicle or the cotyledon leaves had protruded out of the seed and was at least 2 mm long (Bewley and Black, 1994). After each counting, if needed, deionised and autoclave-sterilised water was added to ensure non-limiting moisture. Counting was stopped when no new germination was observed during five consecutive visits. At 5°C, the experiment lasted more than 3 months. Whenever needed, counting was performed in the growth chamber in order to keep the seeds at a constant temperature and a high air humidity.

### Modelling of the Dynamics of Seed Lot Germination

For both the effect of seed deterioration and the genetic diversity, the cumulative percentages of germination values over time were fitted to a modified non-rectangular hyperbole (Thornley et al., 1976), whose parameters have an eco-physiological meaning:

$$y=\left(\frac{1}{2\,\theta }\right)\cdot$$

$$(\alpha \cdot \left(t-t_{c}\right)+y_{\text{max}}-$$

$$\sqrt{{\left(\alpha .\left(t-t_{c}\right)+y_{\text{max}}\right)}^{2}-\left(4.\theta .\alpha .\left(t-t_{c}\right).y_{\text{max}}\right)}).$$

Where *y* is the cumulated seed germination (%); *θ* is a unitless parameter that determines the sharpness of the knee of the curve; *α* is the maximum germination rate (% of germinating seeds per hour); *t* is the time (hour); *t*_*c*_ is the lag to start germination (hour) during which no seed germinates; and *y*_*max*_ is the asymptotic maximum seed germination (%). In order to limit the number of parameters to be estimated, and after several tests, *θ* was fixed to 0.97.

### Statistical Analyses

For the effect of scarification in each of the two temperatures, the observed maximum seed germination (hereafter named as germinability) was submitted to an analysis of variance with the effects of scarification, group, accession within group, repetition, scarification × group and scarification × accession within group. The function *aov* of R was used (R Core Team, 2019).

For the germinability of the two seed lots with high and low germination percentages, a non-linear mixed model (Pinheiro and Bates, 2000) was used to estimate the three parameters (*α*, *t*_*c*_ and *y*_*max*_) and to test the seed lot factor, in Flamande and Gabès separately, at each temperature. The functions *nlsList*, *nlme* and *update* of package *nlme* were sequentially used (Pinheiro et al., 2020) in R.

For the 38 accessions, the non-linear mixed model was similarly used to test the effects of group and accession. A principal component analysis (PCA) was conducted to assess the variability in the three germination parameters of the model in response to temperature (function *PCA* of R).

## Results

### Scarification Effect on the Percentage of Seed Germination

**FIGURE 1:**
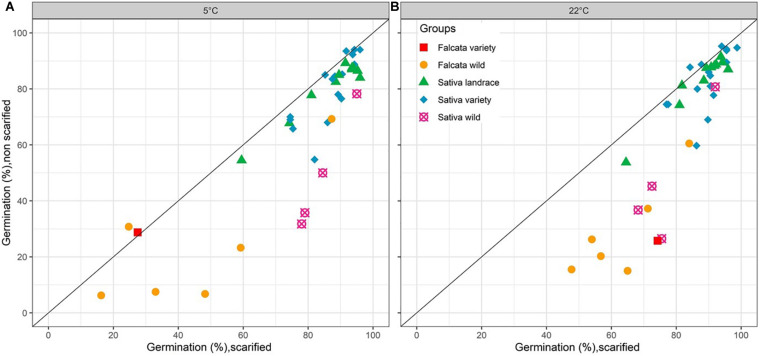
Germinability (observed data, average of 4 biological repetitions) of 38 lucerne populations from 5 different groups with or without scarification at 5°C **(A)** and 22°C **(B)**.

On average for the 38 accessions, scarification had a significant positive effect on germinability at both temperatures 5 and 22°C (Table 2, Figure 1, and Supplementary Table 1). Germinability of non-scarified seeds varied from 6 to 94% at 5°C and from 15 to 95% at 22°C, depending on the accession. With scarification, germinability ranged from 16 to 96% at 5°C and from 15 to 99% at 22°C. The difference of germinability between non-scarified and scarified seeds (Supplementary Table 1) was significant for 12 accessions at both 5 and 22°C, for two other accessions at 5°C only and for three other accessions at 22°C only. This difference reached a maximum of 46 points of percentage at 5°C (Villanueva de Jara, *sativa* wild) and 50 points of percentage at 22°C (Anik, *falcata* variety).

**TABLE 2:** Mean observed germinability over 4 biological repetitions, with or without scarification at 5 and 22°C for the five groups of accessions.

**Group**	**5°C**		**22°C**	
	**Scarified**	**Non-scarified**	**Scarified**	**Non-scarified**
*Falcata* + *glomerata* wild	50.5	27.5	63.1	29.1
*Sativa* wild	84.1	48.9	77.1	47.3
*Falcata* variety	27.5	28.8	74.3	25.8
*Sativa* landrace	86.1	75.3	86.7	77.5
*Sativa* variety	86.8	80.0	88.9	83.0
**Effects of factors in analysis of variance**				
Scarification	572.0***		670.0***	
Group	1585.1***		843.9***	
Accession within group	78.0***		47.0***	
Scarification × group	99.4***		155.2***	
Scarification × accession within group	10.0***		6.0***	
Residual standard error	4.24		4.58	

For the analyses of variance in each temperature, the *F* value and significance are given. *** Significant at *P* < 0.001.

An analysis of variance showed that the effects of group, accession within group, scarification, and their interactions were significant at both temperatures (Table 2 and Figure 1). The scarification effect was positive for the wild accessions, either for *falcata* + *glomerata* (+34 points of percentage) or *sativa* (+30 points of percentage) at 22°C. At 5°C, the figures for the same groups were +23 and +35 points of percentage. For the *falcata* variety, the effect of scarification was large at 22°C (+48 points of percentage) but nil at 5°C, where the germination was very poor without scarification but also with scarification. For the *sativa* landraces and varieties, scarification had a small but significant effect at both temperatures (+7 and +6 points of percentage at 5°C and 22°C, respectively).

### Effect of Seed Lot Deterioration on the Germination Capacity in Response to Temperature

This study was carried out on two landrace accessions, with two seed lots per accession, one with a high germinability and the other with a poor germinability.

**FIGURE 2:**
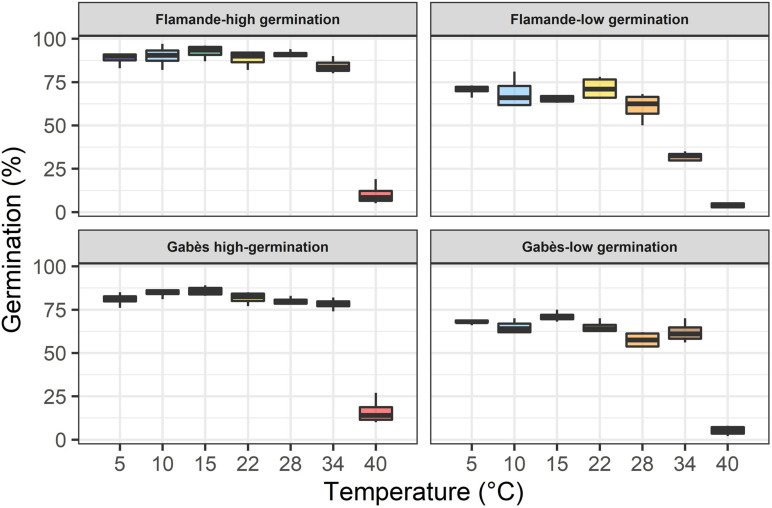
Boxplot showing average germinability (*y*_*max*_ parameter of the model) of two seed lots for two accessions (Flamande and Gabès) in response to temperature.

The seed lots confirmed their expected germinability, either high or low, at all temperatures (Table 3 and Figure 2). The two seed lots of the population Flamande had higher lags to start germination (*t*_*c*_) and lower germination rates (*α*) than the two seed lots of the population Gabès. The low germinability of deteriorated seed lots were associated with low germination rates (*α*; Table 3). At 34°C, the germinability of the deteriorated seed lot, compared to the highly germinating seed lot, was much lower for Flamande than for Gabès.

**TABLE 3:** Germinability (*y*_*max*_ in°C), lag to start germination (*t*_*c*_ in h) and maximum germination rate (*α* in% of germinating seeds per h) for the two seed lots (high and low germination) of two lucerne accessions (Flamande and Gabès) at 7 temperatures.

**Temperature (°C)**	**Parameter**	**Flamande high germ.**	**Flamande low germ.**	**Gabès high germ.**	**Gabès low germ.**
5	α	0.793	0.330 NS	0.806	0.609 NS
	*t*_*c*_	81.5	90.2**	79.6	83.6 NS
	*y*_*max*_	91.2	74.3***	85.6	72.2***
10	α	1.485	0.687*	1.506	0.988**
	*t*_*c*_	28.4	29.2 NS	27.7	26.8 NS
	*y*_*max*_	95.5	72.9***	91.1	68.8***
15	α	3.613	1.516***	4.672	2.188***
	*t*_*c*_	16.0	14.5 NS	13.1	11.1*
	*y*_*max*_	100.3	69.3***	89.6	73.3***
22	α	6.246	2.042**	7.560	4.913*
	*t*_*c*_	12.3	12.3 NS	12.3	11.5*
	*y*_*max*_	94.5	76.2***	85.9	67.6***
28	α	3.591	1.532 NS	7.506	3.035***
	*t*_*c*_	14.0	14.3 NS	12.0	11.0***
	*y*_*max*_	96.7	65.2***	84.1	61.5***
34	α	1.886	0.616 NS	7.421	3.083***
	*t*_*c*_	16.6	16.7 NS	13.3	11.8***
	*y*_*max*_	90.5	33.0***	83.1	66.2***
40	α	0.067	0.090 NS	0.120	0.052***
	*t*_*c*_	51.6	20.1**	4.0	10.6 NS
	*y*_*max*_	11.4	3.7*	17.5	5.7**

For each accession, the significance of the difference between seed lots is given. ***, **, * : significant at *P* < 0.001, *P* < 0.01, *P* < 0.05, respectively. NS: non-significant (*P* > 0.05).

### Genetic Diversity for Germination in Response to Temperature

**FIGURE 3:**
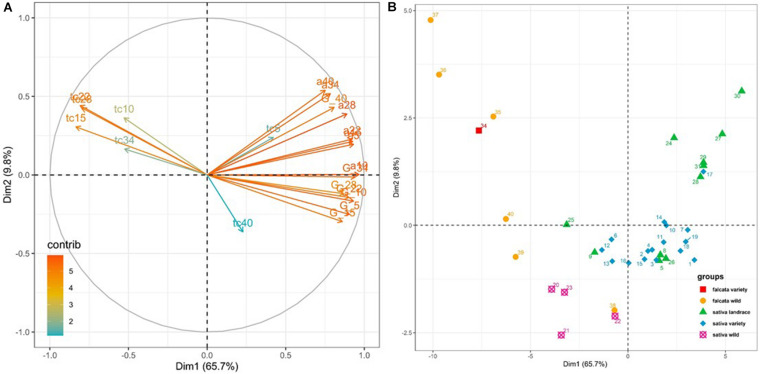
PCA on the parameters of seed germination collected on 38 accessions at 7 temperatures. **(A)** Contribution of the variables* to the first two axes, **(B)** Repartition of the accessions on the first two axes. The codes of the varieties are given in Table 1. *y_*max*_ for germinability, *t*_*c*_ for the lag time to start germination, *a* for the rate of germination. *y*_*max*_, *t*_*c*_ and *a* are followed by the experimental temperature.

A wide diversity for the three parameters of temperature response (germinability, maximum germination rate and lag to start germination) was evidenced among the 38 lucerne accessions (Supplementary Table 2 and Supplementary Figure 1, Figure 3). All parameters at all temperatures shown a significance among accessions (not shown). The effect of group of accessions were also significant except for the maximum germination rates at 5 and 28°C (Table 4).

**TABLE 4:** Germinability (*y*_*max*_ in%), lag to start germination (*t*_*c*_ in h) and maximum germination rate (*α* in% of germinating seeds per h) for the 5 groups of lucerne accessions at 7 temperatures.

**Temperature (°C)**	**Parameter**	***Falcata* + *glomerata* wild**	***Sativa* wild**	***Falcata* variety**	***Sativa* landrace**	***Sativa* variety**	***F* Value**
5	α	0.074	0.109	0.007	0.801	0.701	0.9NS
	*t*_*c*_	77.0	29.6	21.8	80.1	82.3	7.8***
	*y*_*max*_	45.0	81.1	22.8	91.2	90.6	67.2***
10	α	0.346	0.832	0.062	1.547	1.440	11.2***
	*t*_*c*_	33.8	29.6	72.5	28.0	28.4	7.2***
	*y*_*max*_	65.3	83.5	74.5	95.1	95.2	43.5***
15	α	0.797	1.938	0.660	4.271	3.395	22.2***
	*t*_*c*_	22.0	15.9	28.1	14.4	15.1	29.2***
	*y*_*max*_	71.0	88.9	75.9	93.3	95.6	31.7***
22	α	0.673	1.820	0.427	6.407	5.122	4.1***
	*t*_*c*_	32.8	13.6	27.8	12.3	12.4	67.3***
	*y*_*max*_	68.6	78.9	80.7	92.6	94.7	33.4***
28	α	0.479	0.784	0.142	5.415	3.803	1.5NS
	*t*_*c*_	61.9	16.4	74.7	12.6	13.0	151.1***
	*y*_*max*_	63.3	76.2	72.1	90.3	92.8	20.9***
34	α	0.159	0.155	0.019	3.601	2.066	15.4***
	*t*_*c*_	41.2	25.6	17.8	15.2	15.7	8.9***
	*y*_*max*_	20.1	28.0	17.4	83.8	84.3	7.0***
40	α	0.024	0.013	0.007	0.131	0.091	23.5***
	*t*_*c*_	49.1	36.7	26.9	31.0	41.6	39.3***
	*y*_*max*_	3.5	5.1	3.1	18.6	15.4	42.6***

The *F* value is for the test of difference among groups. *** significant at *P* < 0.001, NS: non-significant at *P* = 0.05.

In the PCA analysis performed with the values of germinability (*y*_*max*_) and the estimated values of maximum germination rate (*α*) and lag to start germination (*t*_*c*_) estimated at seven temperatures (5, 10, 15, 22, 28, 34, 40°C; Figure 3), the first two axes were significant (eigenvalue > 1) and contributed for 75.5% of total variance (Supplementary Table 3 and Figure 3A). Axis 1 explained 65.7% of the total variability and was mainly correlated to germination rates at 10, 5 and 15°C and germinability at 10 and 34°C (Figures 3A,B and Supplementary Table 2). Axis 2, which explained 9.8% of the total variability, was mainly correlated to the germination rates at 40 and 34°C (Supplementary Table 3). Germinability at all temperatures was positively correlated with maximum germination rate at all temperatures (Figure 3A). Lag to start germination from 10 to 34°C was negatively correlated with both germinability and maximum germination rate (Figure 3A). As a whole, the accessions that had high germinability germinated shortly and quickly after seed imbibition.

All germination parameters were optimum at 15 or 22°C for all groups of accessions (Table 4 and Supplementary Figure 1). With progressing colder temperatures, the lag to start germination increased and the germination rate decreased for all groups of accessions, while germinability decreased for the *falcata* and *glomerata* accessions only. With progressing higher temperatures, the germinability of the cultivated *sativa* accessions remained high up to 34°C, while their lag to start germination increased and their germination rate decreased. At 40°C, all germination parameters of the cultivated *sativa* accessions were altered. For the wild accessions and the *falcata* variety, germinability was still high at 28°C, but not at 34°C nor at 40°C, and lag to start germination and germination rate were strongly modified. Figure 3B demonstrates that the groups behaved in a different way in the PCA. The accessions of both ssp *sativa* landrace and ssp *sativa* variety groups showed a high germinability as well as a high germination rate and a small lag to start germination. This means that these cultivated accessions are able to germinate shortly after the conditions are adequate and that all the seeds germinate at once. Moreover, it is to be noted that, as shown from their position on axis 2 of the PCA, the ssp *sativa* accessions with an autumn dormancy between 3 and 7 had a lower germination rate at 34°C than the non-dormant accessions with dormancy 9 which originate from Tunisia, Morocco or Iran (Figure 3B). Compared to the cultivated *sativa* accessions, accessions of the wild *falcata* and *glomerata* group showed a low germinability and very low germination rate as well as a high lag to start germination. The *falcata* variety (Anik, no 34) also showed a low germinability, a low germination rate and a long lag to start germination. However, the ssp *falcata* accession Krasnokutskaya (no 38) from Russia showed a higher germinability, a higher germination rate and a lower lag to start germination than the other accessions of the ssp *falcata* wild group (Figure 3B). The wild *sativa* accessions that originated from Spain had a higher germinability and a higher germination rate than the wild *falcata* accessions. Interestingly, the wild accessions that originated from Spain had only slightly lower germination rate and lag to start germination than cultivated *sativa* accessions from 5 to 22°C, but all their germination parameters were strongly altered at 34°C, with a low germinability, a long lag to start germination and a slow germination rate (Table 3). At 5°C, the germinability of the *sativa* accessions, whether wild or cultivated, were higher than that of the *falcata* and *glomerata* accessions.

## Discussion

This study aims at demonstrating that lucerne seed germination is influenced by physical treatment (scarification), seed lot deterioration and genetic diversity in response to temperature. It also illustrates the interaction between these factors and gives insights about the history of domestication and selection.

### Scarification Effect on the Percentage of Seed Germination

Physical seed dormancy caused by the water-impermeable seed coat that inhibits water absorption is well described in many species (Baskin and Baskin, 2004, 2014), including legumes (Taylor, 2005; Smykal et al., 2014). Scarification, by a physical or chemical alteration of the seed coat, increases seed germination. Our results on 38 accessions of lucerne indicate that, on average, non-scarified seeds of lucerne had a lower germinability than scarified seeds at both temperatures (5 and 22°C). This scarification effect on germination response depended on accessions. It was higher for the wild accessions, either *falcata* or *sativa* ssp. than for the cultivated accessions. A large effect of mechanical scarification had previously been reported on one naturalised American yellow-flowered population (Narem, 2009). This could mean that the domestication syndrome which includes germination, well described on legumes (Smykal et al., 2014), also applies to lucerne. Our results suggest that the seed dormancy mechanisms carried by wild accessions have been lost during domestication and breeding. This loss enables the seeds of cultivated accessions to germinate as soon as they are sown, even if severe stress conditions (frost, drought) could occur shortly afterwards. It is thus the responsibility of the farmer to choose an adequate sowing season to ensure good establishment and favourable growing conditions. In the wild, germination is submitted to the random occurrence of seed coat wound, thereby spreading germination over a long time period after the seeds reach the soil. As other domestication traits whose inheritance is usually simple, a small number of genes or a single gene probably govern the susceptibility of lucerne seed germination to scarification (Smykal et al., 2014). This domestication trait comes in addition to the domestication syndrome already described in lucerne: plant vigour caused by polyploidy and erect growth habit to increase forage production and facilitate harvesting, and coiled-shape pods to limit seed shattering (Annicchiarico et al., 2015). The early farmers have probably selected plants that do not require scarification, by sampling the first emerged seedlings after sowing, and modern breeders proceed similarly. However, in our study as in others (Acharya et al., 1999; Palfi, 2007; Kandil et al., 2012), the germination of cultivated varieties was enhanced by scarification, which means that the loss of the hardseededness trait is not yet completed. Either the genetics of lucerne (autotetraploidy and allogamy) make the removal of this trait from the breeding populations difficult, or the hardseededness is under a quantitative genetic control that implies many genes (Finkelstein et al., 2008). The germination of the single *falcata* variety studied here was strongly enhanced by scarification. This variety has been bred for adaptation to Northern Canada from a wild Russian *falcata* population (Pankiw and Siemens, 1976) but germination without scarification has probably not been taken into account. In all situations, either with elite breeding pools or in breeding programs that include wild material, further selection devoted to this trait should improve germination and thus lucerne establishment.

### Effect of Seed Deterioration

For both landraces, Flamande and Gabès, the low germination seed lot germinated less, with lower germination rate and longer lag to start germination than the high germination seed lot, at all temperatures. The differences between the two seed lots were higher at extreme temperatures than at average temperatures (15–22°C). This effect of temperature on the germination of deteriorated seed lots has little been studied. On pea (*Pisum sativum*) and bean (*Phaseolus vulgaris*), for example, all the seed lots of all genotypes had a high germinability, even at extreme temperatures, except for two bean seed lots at 10°C (Raveneau et al., 2011). For pea seeds produced under accelerated desiccation conditions, germination at a cold temperature (5°C) was only reduced by 10% compared to a control seed lot (Raveneau, 2012). The results obtained here suggest that the seeds with altered germinability should be sown in optimal temperature conditions, either for culture at the farm level or for multiplication in Genetic Resources Centres.

### Genetic Diversity

The impact of temperature on germination is well established and cardinal temperatures have been determined on many species, such as weeds (Long et al., 2015), crops (Durr et al., 2015) and cover crops (Tribouillois et al., 2016). All accessions evaluated in our work exhibited the highest germinability and rate, as well as the shortest lag to start germination at 15°C or 22°C. The optimal constant temperature for lucerne germination is thus within this range. As discussed below, the germination was not as good at colder or warmer temperatures but the effect of extreme temperatures depended on the accessions.

The within-species diversity for germination in response to temperature has received little attention either in the number of accessions or in the ranges of temperatures that have been tested. In our study, we analysed 38 accessions from the whole *M. sativa* complex, with diploid and tetraploid accessions of wild and cultivated sub-species. This range of diversity is interesting because subspecies freely intercross at a ploidy level. Wild populations that are under-represented in breeding pools (Muller et al., 2006) could be used to integrate some traits for adaptation to new constraints, such as climate change, or new practices, such as cover crops (Labreuche, 2017; Moore et al., 2019). These accessions were studied in seven temperatures ranging from 5°C, a temperature which mimics cold autumns or springs, to a temperature of 40°C, which could occur with late spring sowings under warm climates. In our study, germinability was very low, on average 12% only, at 40°C. In contrast, germinability at 40°C ranged from 60 to 80% in a preliminary study on a subset of seven accessions among the ones studied here (Ahmed et al., 2019). We suppose that the high germinability in the latter study originates both from the temperature during counting (counting in the laboratory at 20°C) and/or from the natural light received by the seeds during counting (L. Ahmed, personal observation).

Within cultivated *sativa* accessions, germinability varied little at temperatures from 5 to 34°C. Breeding for stable germination is probably conducted, even unconsciously, when the first emerged plantlets are selected to constitute the pool of plants to be studied or crossed. In the field too, the first emerged plants gain a competitive advantage on the later emerged ones because they capture resources such as light. Indeed, there is no difference between landraces and varieties, meaning that this selection has been operating for a long time. Other studies have already established this low range of genetic diversity among varieties, but with experiments most frequently conducted under temperatures between 10 and 30°C only (Mcelgunn, 1973; Hampton et al., 1987; Brar et al., 1991; Butler et al., 2014). Anyway, the non-dormant cultivated accessions (dormancy 9, from Tunisia, Morocco and Iran) had higher germination rates at 34°C than the more dormant accessions (dormancy between 3 and 7). Non-dormant accessions are bred and used under warm climates in which hot periods may occur in the sowing time, either autumn or spring. This inability to germinate at high temperature has probably been selected as a global adaptation to local conditions.

The wild accessions, on average, had a lower germinability, a lower germination rate and a longer lag to start germination than cultivated accessions. Moreover, wild accessions adapted to cold conditions (*falcata* ssp) had a low germinability at 5°C and wild accessions from Spain (*sativa* ssp) had a low germination rate at 34°C but a high germination rate at 5°C. This counterintuitive result can easily be explained: if the falcata accessions germinated at 5°C (in the autumn), they would have a large risk of encountering cold conditions in the following weeks. It is an adaptation for populations that their seeds germinate when temperatures reach at least 10°C. Reversely, if wild accessions from Spain germinated at 34°C (in the spring), they would suffer from associated dry conditions. In the case where they germinate at 5°C, this cold temperature is statistically not followed by colder temperatures, so establishment is preserved. These adaptations are ways to escape harsh (either cold or dry) conditions in the wild. In our study, the *falcata* variety had germination characteristics close to those of wild *falcata*. Its breeding, from a wild *falcata* accession (Pankiw and Siemens, 1976), probably did not include a selection for germination at low temperatures, as seen above for scarification. Krasnokutskaya, from Russia, received as a wild *falcata* accession by the Genetic Resources Centre of INRAE, Lusignan, France, showed germination characteristics close to those of cultivated *sativa* accessions, but its lag to start germination at 34°C was longer. This germination ability suggests that Krasnokutskaya is probably a cultivated accession selected for improved establishment. Another study (Julier et al., 1995) revealed that this accession exhibited a relative erect growth habit, long stems and large seeds, which are relevant breeding traits.

This study has been conducted with seed lots that have been stored for different lengths of time in cold chambers. With this allogamous insect-pollinated species, the seed production of many accessions in the same location and year is challenging. The storage duration may impact seed germination response to temperature but we suppose that this impact is smaller than the genetic difference among accessions. Indeed, these results show that natural selection for germination in response to temperature has shaped species diversity. The mean value of this trait, which can be considered as an adaptative trait, has changed during the evolution of the *Medicago sativa* complex in several subspecies adapted to different regions and climates. In addition, human selection has been efficient to alter the effect of temperature on germination, as also demonstrated in experimental work (Klos and Brummer, 2000). In breeding programmes, a good germination in a large range of temperatures is required. When the plant material originates from cultivated pools, no more selection is needed. When wild accessions are introduced in a breeding pool, attention must be paid to reach a high germination, especially at extreme temperatures.

## Data Availability Statement

The datasets presented in this study can be found in online repositories. The names of the repository/repositories and accession number(s) can be found below: https://doi.org/10.15454/YIECZS.

## Author Contributions

AE-G and BJ conceived the study. AE-G, LA, and BJ designed the experiment. LA collected the data with contributions of BJ, AE-G, and WG. BJ, WG, and LA carried out the data analysis. All co-authors contributed to wrote the manuscript. All authors contributed to the article and approved the submitted version.

## Conflict of Interest

The authors declare that the research was conducted in the absence of any commercial or financial relationships that could be construed as a potential conflict of interest.
