# Supplementary material for: The History of Domestication and Selection of Lucerne: A New Perspective From the Genetic Diversity for Seed Germination in Response to Temperature and Scarification
Source: Front Plant Sci. 2021 Jan 21;11:578121. doi: 10.3389/fpls.2020.578121 (PMC7860617; doi:10.3389/fpls.2020.578121)

# Supplementary Figure S1 | Average germinability of 38 lucerne accessions in response to temperature (boxplot on observed data).


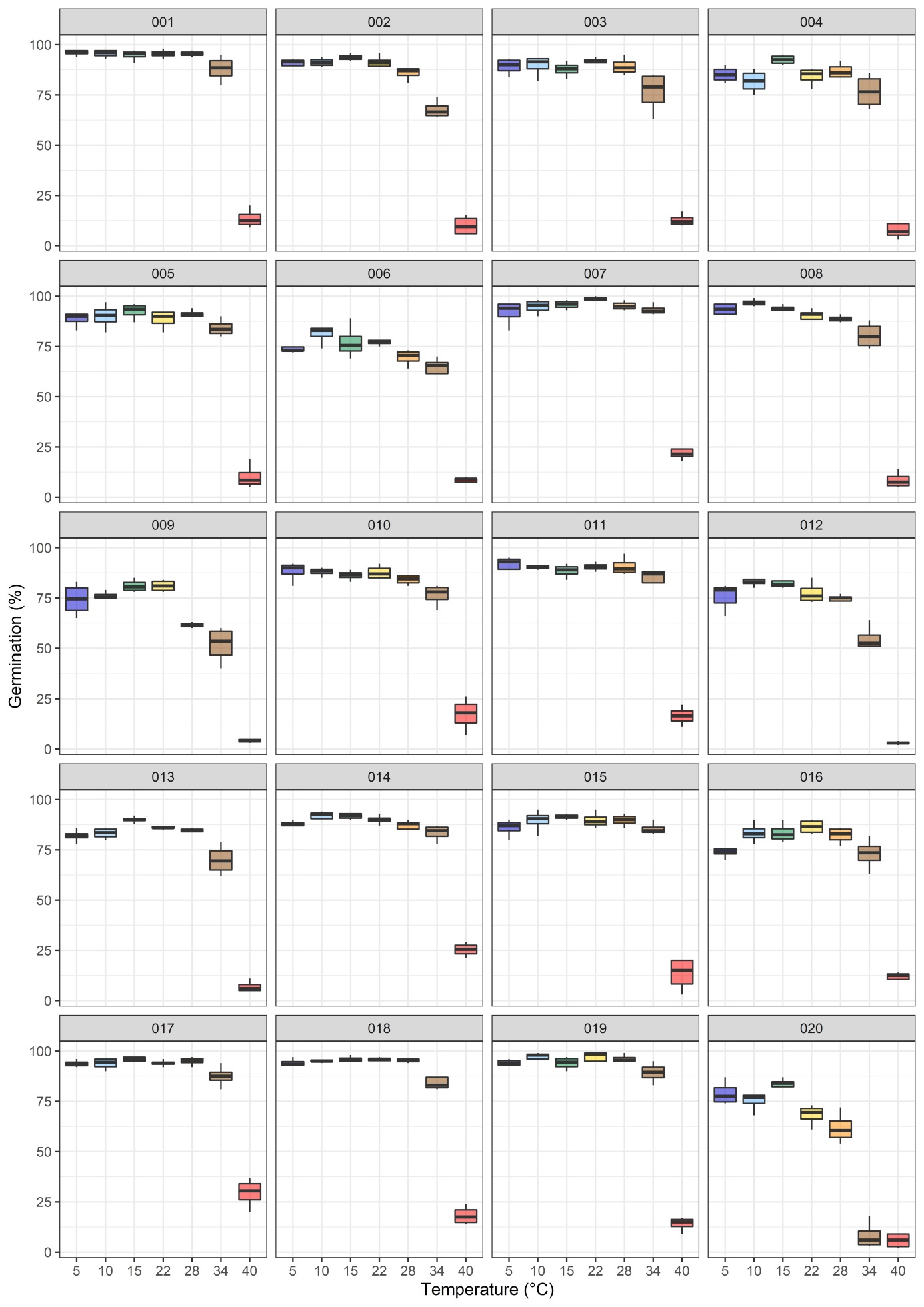


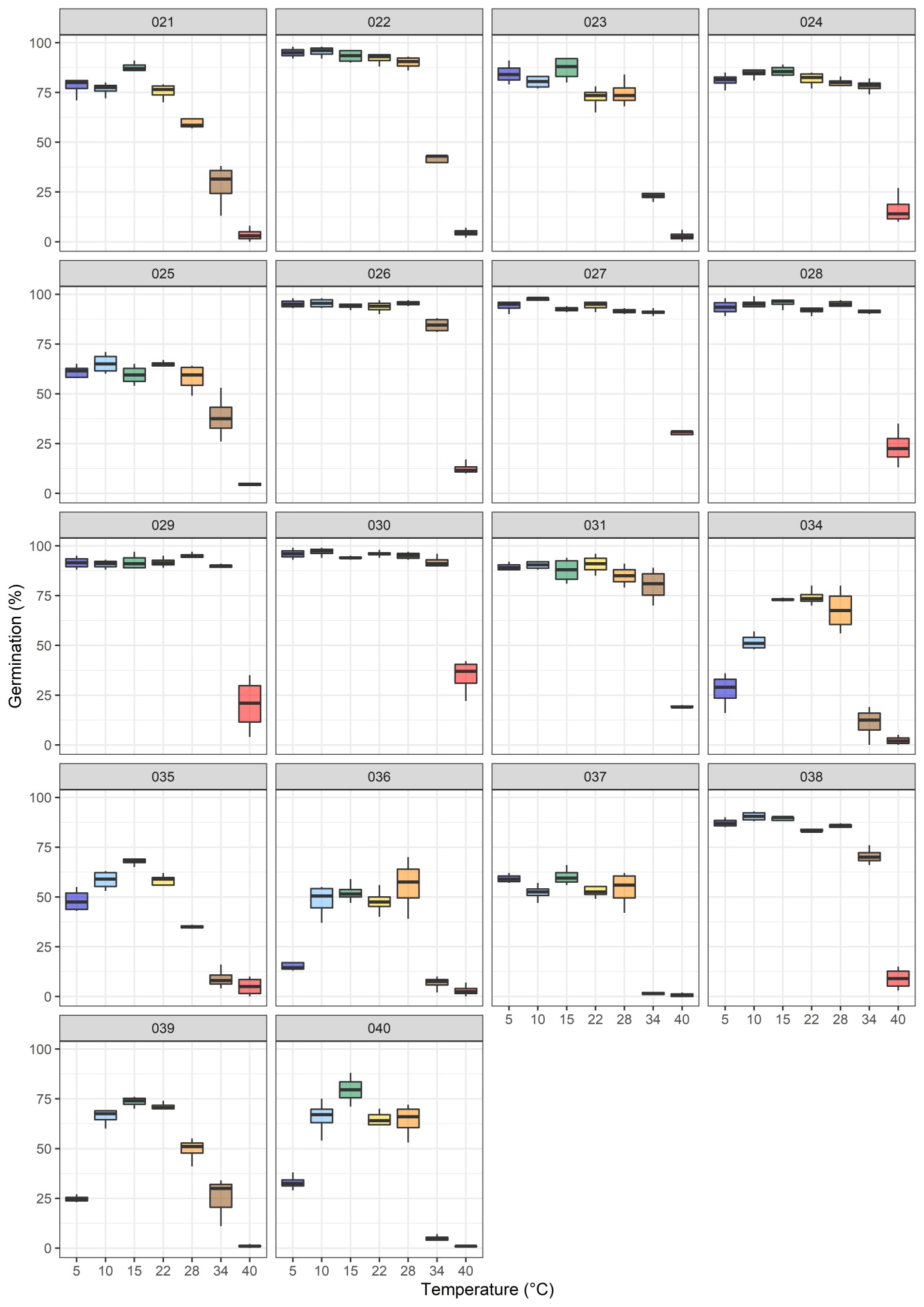

Supplement: Supplementary file 1 [file Data_Sheet_1.zip › Figure 1.docx]
