# Supplementary material for: The History of Domestication and Selection of Lucerne: A New Perspective From the Genetic Diversity for Seed Germination in Response to Temperature and Scarification
Source: Front Plant Sci. 2021 Jan 21;11:578121. doi: 10.3389/fpls.2020.578121 (PMC7860617; doi:10.3389/fpls.2020.578121)
Supplement: Supplementary file 1 [file Data_Sheet_1.zip › Table 1.docx]

# Supplementary Table S1 | Mean germinability with or without scarification at 5 and 22°C for the 38 accessions. For each accession at each temperature, the germinability is different when followed by two different letters.

|  |  |  | **5°C** | | **22°C** | |
| --- | --- | --- | --- | --- | --- | --- |
| Code | **Name** | **Group** | **Scarified** | **Non scarified** | **Scarified** | **Non scarified** |
| 035 | Romanica | *falcata* wild | 48.25 a | 6.75 b | 56.75 a | 20.25 b |
| 036 | Quasifalcata | *falcata* wild | 16.25 a | 6.25 a | 47.75 a | 15.50 b |
| 038 | Krasnokutskaya | *falcata* wild | 87.25 a | 69.25 b | 84.00 a | 60.50 b |
| 039 | Maron | *falcata* wild | 24.75 a | 30.75 b | 71.25 a | 37.25 b |
| 040 | Malzeville | *falcata* wild | 33.00 a | 7.50 b | 65.00 a | 15.00 b |
| 037 | Glomerata | *glomerata* wild | 59.25 a | 23.25 b | 54.00 a | 26.25 b |
| 034 | Anik | *falcata* variety | 27.50 a | 28.75 a | 74.25 a | 25.75 b |
| 020 | Monte Oscuro | *sativa* wild | 79.00 a | 35.75 b | 68.25 a | 36.75 b |
| 021 | Villanueva de Jara | *sativa* wild | 78.00 a | 31.75 b | 75.50 a | 26.50 b |
| 022 | Villamajor | *sativa* wild | 95.00 a | 78.25 b | 92.00 a | 80.75 b |
| 023 | Pancrudo | *sativa* wild | 84.50 a | 50.00 b | 72.50 a | 45.25 b |
| 005 | Flamande* | *sativa* landrace | 88.50 a | 82.50 a | 88.50 a | 83.00 a |
| 008 | Poitou | *sativa* landrace | 93.50 a | 87.00 a | 89.25 a | 87.50 a |
| 009 | Provence | *sativa* landrace | 74.25 a | 67.75 a | 81.00 a | 74.25 a |
| 024 | Gabès* | *sativa* landrace | 81.00 a | 77.75 a | 81.75 a | 81.25 a |
| 025 | Cremonese | *sativa* landrace | 59.50 a | 54.50 a | 64.50 a | 53.75 a |
| 026 | Crau | *sativa* landrace | 95.25 a | 86.50 b | 93.75 a | 91.50 a |
| 027 | Demnate3 | *sativa* landrace | 94.00 a | 87.50 a | 94.25 a | 89.50 a |
| 028 | Dra15 | *sativa* landrace | 93.50 a | 87.50 a | 92.25 a | 88.75 a |
| 029 | Atlas | *sativa* landrace | 91.50 a | 89.25 a | 91.75 a | 88.25 a |
| 030 | Ziz10 | *sativa* landrace | 96.00 a | 84.00 b | 96.00 a | 87.00 b |
| 031 | Baghdadi | *sativa* landrace | 89.50 a | 85.00 a | 90.75 a | 87.75 a |
| 001 | Banat VS | *sativa* variety | 96.00 a | 94.00 a | 95.50 a | 93.75 a |
| 002 | SW Nexus | *sativa* variety | 90.25 a | 76.50 a | 90.50 a | 81.00 a |
| 003 | Luzelle | *sativa* variety | 89.25 a | 78.00 a | 91.50 a | 77.75 b |
| 004 | Holyna | *sativa* variety | 85.25 a | 85.00 a | 84.25 a | 87.75 a |
| 006 | Lukal | *sativa* variety | 74.50 a | 70.00 a | 77.00 a | 74.50 a |
| 007 | Ludelis | *sativa* variety | 91.75 a | 93.50 a | 98.75 a | 94.75 a |
| 010 | Barmed | *sativa* variety | 88.25 a | 84.50 a | 87.75 a | 88.75 a |
| 011 | Harpe | *sativa* variety | 90.50 a | 85.25 a | 90.50 a | 84.75 a |
| 012 | Orca | *sativa* variety | 75.33 a | 65.75 a | 77.50 a | 74.50 a |
| 013 | Radius | *sativa* variety | 82.00 a | 54.75 b | 86.25 a | 59.75 b |
| 014 | FG-CO416C4164 | *sativa* variety | 87.50 a | 83.50 a | 90.00 a | 86.00 a |
| 015 | Alforex6 | *sativa* variety | 86.00 a | 68.00 b | 89.75 a | 69.00 b |
| 016 | Gongnong1 | *sativa* variety | 74.50 a | 69.00 a | 86.50 a | 80.00 a |
| 017 | Magna 790 | *sativa* variety | 93.75 a | 92.25 a | 94.00 a | 95.25 a |
| 018 | Bauding | *sativa* variety | 94.25 a | 88.75 b | 95.50 a | 89.50 a |
| 019 | Picena GR | *sativa* variety | 94.25 a | 94.00 a | 95.50 a | 94.25 a |
