# Supplementary material for: The History of Domestication and Selection of Lucerne: A New Perspective From the Genetic Diversity for Seed Germination in Response to Temperature and Scarification
Source: Front Plant Sci. 2021 Jan 21;11:578121. doi: 10.3389/fpls.2020.578121 (PMC7860617; doi:10.3389/fpls.2020.578121)
Supplement: Supplementary file 1 [file Data_Sheet_1.zip › Table 2.docx]

# Supplementary Table S2 | Germinability (*ymax* in %) and maximum germination rate (*α* in % of germinating seeds per h) for the 38 lucerne accessions at some temperatures. The 9 most significant variables of axis 1 and the most significant variable of axis 2 in the PCA are shown.

| **Code** | **Name** | **Group** | *α28* | *ymax22* | *ymax5* | *α22* | *α15* | *α5* | *ymax10* | *ymax34* | *α10* | *α40* |
| --- | --- | --- | --- | --- | --- | --- | --- | --- | --- | --- | --- | --- |
| **035** | Romanica | *falcata* wild | 0.169 | 61.3 | 52.8 | 0.422 | 0.690 | 0.036 | 61.8 | 8.9 | 0.256 | 0.059 |
| **036** | Quasifalcata | *falcata* wild | 0.111 | 51.0 | 15.5 | 0.304 | 0.351 | 0.017 | 50.6 | 6.2 | 0.168 | 0.034 |
| **038** | Krasnokutskaya | *falcata* wild | 2.262 | 88.3 | 84.5 | 2.235 | 1.949 | 0.209 | 94.3 | 76.8 | 0.912 | 0.039 |
| **039** | Maron | *falcata* wild | 0.185 | 75.5 | 21.6 | 0.536 | 0.721 | 0.022 | 67.4 | 23.7 | 0.238 | 0.005 |
| **040** | Malzeville | *falcata* wild | 0.121 | 70.1 | 32.2 | 0.386 | 0.609 | 0.022 | 63.7 | 3.8 | 0.143 | 0.005 |
| **037** | Glomerata | *falcata* wild | 0.026 | 65.4 | 63.2 | 0.156 | 0.462 | 0.136 | 54.3 | 1.0 | 0.362 | 0.002 |
| **034** | Anik | *falcata* variety | 0.142 | 80.7 | 22.8 | 0.427 | 0.660 | 0.007 | 74.5 | 17.4 | 0.062 | 0.007 |
| **020** | Monte Oscuro | *sativa* wild | 0.394 | 69.7 | 76.6 | 1.077 | 1.182 | 0.088 | 77.3 | 8.6 | 0.523 | 0.014 |
| **021** | Villanueva de Jara | *sativa* wild | 0.496 | 75.3 | 76.8 | 1.258 | 1.173 | 0.052 | 75.3 | 32.0 | 0.664 | 0.016 |
| **022** | Villamajor | *sativa* wild | 1.261 | 96.8 | 86.0 | 3.171 | 3.320 | 0.229 | 99.8 | 45.4 | 1.354 | 0.015 |
| **023** | Pancrudo | *sativa* wild | 0.984 | 73.7 | 85.0 | 1.772 | 2.079 | 0.066 | 81.8 | 26.1 | 0.786 | 0.008 |
| **005** | Flamande | *sativa* landrace | 3.590 | 94.5 | 91.0 | 6.148 | 3.615 | 0.768 | 95.4 | 90.5 | 1.503 | 0.061 |
| **008** | Poitou | *sativa* landrace | 3.605 | 94.3 | 97.2 | 5.250 | 3.732 | 0.836 | 103.4 | 87.1 | 1.666 | 0.069 |
| **009** | Provence | *sativa* landrace | 1.412 | 85.0 | 79.4 | 2.933 | 2.468 | 0.383 | 81.9 | 56.5 | 0.947 | 0.051 |
| **024** | Gabès | *sativa* landrace | 7.509 | 85.9 | 85.6 | 7.447 | 4.849 | 0.777 | 91.1 | 82.5 | 1.516 | 0.104 |
| **025** | Cremonese | *sativa* landrace | 1.556 | 68.3 | 65.3 | 2.402 | 2.091 | 0.383 | 69.8 | 42.1 | 0.870 | 0.050 |
| **026** | Crau | *sativa* landrace | 3.314 | 100.0 | 98.7 | 5.300 | 3.330 | 0.880 | 102.5 | 91.6 | 1.559 | 0.084 |
| **027** | Demnate3 | *sativa* landrace | 7.119 | 99.9 | 98.5 | 8.819 | 5.062 | 1.145 | 104.3 | 96.7 | 1.883 | 0.219 |
| **028** | Dra15 | *sativa* landrace | 6.691 | 97.6 | 97.6 | 7.761 | 4.581 | 0.804 | 100.8 | 97.2 | 1.727 | 0.163 |
| **029** | Atlas | *sativa* landrace | 7.906 | 97.3 | 96.5 | 6.819 | 4.899 | 0.849 | 97.7 | 95.2 | 1.694 | 0.158 |
| **030** | ZIZ10 | *sativa* landrace | 9.055 | 100.4 | 100.3 | 9.354 | 6.033 | 1.005 | 104.0 | 97.2 | 1.869 | 0.291 |
| **031** | Baghdadi | *sativa* landrace | 7.809 | 96.0 | 93.3 | 8.249 | 6.325 | 0.975 | 95.8 | 85.1 | 1.783 | 0.196 |
| **001** | Banat VS | *sativa* variety | 4.747 | 101.9 | 100.4 | 8.676 | 4.860 | 1.120 | 102.8 | 95.0 | 1.828 | 0.069 |
| **002** | Sw Nexus | *sativa* variety | 3.686 | 96.0 | 93.7 | 4.688 | 3.069 | 0.601 | 97.4 | 73.0 | 1.201 | 0.086 |
| **003** | Luzelle | *sativa* variety | 3.809 | 97.3 | 92.3 | 5.224 | 3.599 | 0.675 | 95.8 | 82.6 | 1.617 | 0.072 |
| **004** | Holyna | *sativa* variety | 4.029 | 89.2 | 88.4 | 5.552 | 4.178 | 0.650 | 87.1 | 83.1 | 1.519 | 0.066 |
| **006** | Lukal | *sativa* variety | 2.804 | 81.8 | 78.9 | 3.700 | 2.928 | 0.461 | 86.6 | 68.2 | 1.275 | 0.046 |
| **007** | Ludelis | *sativa* variety | 4.630 | 105.7 | 95.6 | 5.305 | 3.922 | 0.881 | 102.1 | 99.7 | 1.635 | 0.123 |
| **010** | Barmed | *sativa* variety | 4.000 | 92.1 | 92.2 | 6.449 | 3.600 | 0.870 | 94.5 | 82.1 | 1.569 | 0.117 |
| **011** | Harpe | *sativa* variety | 4.216 | 94.9 | 94.0 | 5.825 | 3.547 | 0.741 | 96.4 | 89.6 | 1.541 | 0.078 |
| **012** | Orca | *sativa* variety | 2.440 | 82.4 | 78.0 | 3.973 | 2.158 | 0.416 | 89.1 | 59.6 | 0.962 | 0.024 |
| **013** | Radius | *sativa* variety | 2.239 | 89.3 | 82.3 | 2.681 | 2.183 | 0.398 | 86.4 | 74.9 | 0.909 | 0.039 |
| **014** | FG-CO416C4164 | *sativa* variety | 3.449 | 94.6 | 92.0 | 4.457 | 2.689 | 0.723 | 96.5 | 89.6 | 1.470 | 0.140 |
| **015** | Afxh144110 | *sativa* variety | 3.283 | 94.8 | 89.4 | 3.143 | 2.171 | 0.545 | 94.7 | 92.3 | 1.085 | 0.093 |
| **016** | Gongnong1 | *sativa* variety | 3.222 | 92.3 | 75.1 | 3.365 | 2.729 | 0.505 | 89.3 | 77.6 | 1.332 | 0.066 |
| **017** | Magna 790 | *sativa* variety | 5.485 | 100.5 | 98.7 | 5.212 | 4.757 | 0.913 | 100.7 | 93.6 | 1.620 | 0.246 |
| **018** | Bauding | *sativa* variety | 4.260 | 101.1 | 97.8 | 6.164 | 3.717 | 0.804 | 101.6 | 92.3 | 1.661 | 0.109 |
| **019** | Picena GR | *sativa* variety | 4.547 | 101.9 | 100.1 | 7.544 | 4.204 | 0.908 | 102.1 | 96.1 | 1.825 | 0.079 |
