# Supplementary material for: The History of Domestication and Selection of Lucerne: A New Perspective From the Genetic Diversity for Seed Germination in Response to Temperature and Scarification
Source: Front Plant Sci. 2021 Jan 21;11:578121. doi: 10.3389/fpls.2020.578121 (PMC7860617; doi:10.3389/fpls.2020.578121)
Supplement: Supplementary file 1 [file Data_Sheet_1.zip › Table 3.docx]

# Supplementary Table S3. Eigenvalue and percentage of variance for the first two axes (PC1 and PC2) derived from principal component analysis (PCA) on 38 lucerne accessions and correlation between the axes and the variables (*y_max_*: germinability, *t_c_*: lag to start germination, *α*: maximum germination rate) at 7 temperatures.

| Variable | PC1 | PC2 |
| --- | --- | --- |
| Eigenvalue | 13.8 | 2.06 |
| variance | 65.7% | 9.8% |
|  |  |  |
| *α5* | 0.932 | 0.195 |
| *t_c_5* | 0.421 | 0.239 |
| *ymax5* | 0.907 | -0.251 |
| *α10* | 0.967 | 0.003 |
| *t_c_10* | -0.528 | 0.363 |
| *ymax10* | 0.932 | -0.167 |
| *α15* | 0.927 | 0.214 |
| *t_c_15* | -0.834 | 0.306 |
| *ymax15* | 0.861 | -0.297 |
| *α22* | 0.922 | 0.228 |
| *t_c_22* | -0.808 | 0.442 |
| *ymax22* | 0.903 | -0.144 |
| *α28* | 0.890 | 0.388 |
| *t_c_28* | -0.796 | 0.426 |
| *ymax28* | 0.870 | -0.120 |
| *α34* | 0.785 | 0.517 |
| *t_c_34* | -0.525 | 0.165 |
| *ymax34* | 0.943 | -0.013 |
| *α40* | 0.752 | 0.539 |
| *t_c_40* | 0.228 | -0.363 |
| *ymax40* | 0.808 | 0.430 |
